# Triangulating perspectives on functional neuroimaging for disorders of mental health

**DOI:** 10.1186/1471-244X-13-208

**Published:** 2013-08-08

**Authors:** James A Anderson, Ania Mizgalewicz, Judy Illes

**Affiliations:** 1 Division of Neurology, Department of Medicine, National Core for Neuroethics, University of British Columbia, Vancouver, British Columbia, Canada

## Abstract

**Background:**

Functional neuroimaging is being used in clinical psychiatry today despite the vigorous objections of many in the research community over issues of readiness. To date, a systematic examination of the perspectives of key stakeholders involved in this debate has not yet been attempted. To this fill this gap, we interviewed investigators who conduct functional neuroimaging studies involving adults with mood disorders, schizophrenia, obsessive compulsive disorder, and/or attention deficit hyperactivity disorder, providers who offer clinical neuroimaging services in the open marketplace, and consumers of these services, in order to understand perspectives underlying different views and practices.

**Methods:**

Semi-structured interviews were conducted over the telephone. Verbal consent was obtained and all interviews were audio recorded. Interviews of investigators and service providers followed the same interview guide. A separate set of questions was developed for consumers. All interviews were transcribed and made software ready. We applied the qualitative methodology of constant comparison to analyze the data, whereby two researchers independently analyzed the results into textual themes. Coding discrepancies were discussed until consensus was achieved.

**Results:**

Investigators, service providers, and consumers held many common perspectives about the potential or actual risks and benefits of functional neuroimaging for mental illness. However, we also found striking divergences. Service providers focused on the challenges posed by the persistence of symptoms based diagnostic categories, whereas the limitations of the science in this area was the challenge noted most frequently by investigators. The majority of consumers stated that their expectations were met.

**Conclusion:**

Our findings point toward a fundamental tension between academic investigators on the one hand, and commercial service providers and their customers on the other. This scenario poses dangers to the communities directly involved, and to public trust in science and medicine more generally. We conclude with recommendations for work that needs to be done to minimize tensions and maximize the potential of neurotechnology through concerted efforts to respect its limitations while leveraging the strengths, investments, and hopes of each stakeholder group.

## Background

Millions of people worldwide are affected by mental disorders such as depression, bipolar disorder, schizophrenia, post-traumatic stress disorder and anxiety disorders. In 2011, the World Health Organization reported that disorders of mental health are the leading causes of disability adjusted life years worldwide, accounting for 37% of healthy years lost from non-communicable diseases [1]. A recent report by the World Economic Forum estimated the global cost of mental illness at nearly USD $2.5 trillion in 2010, with a projected increase to over USD $6 trillion by 2030 [2]. In the USA, an estimated 11 million American adults (approximately 5% of all adults) suffer from a seriously disabling mental illness [3]. The indirect and direct costs associated with mental illness in the USA have been estimated to be $300 billion annually [4]. The projected budget for the USA-based National Institute of Mental Health (NIMH) – the largest funder of mental health research in the world – was $1,479,204,000 [5], with more than two thirds of that budget slated for research that aims to improve the understanding of the fundamental biology of mental disorders, and the translation of those findings into improved prevention, diagnosis, treatment selection and monitoring [5]. Research studies employing various neuroimaging technologies consume a significant portion of this budget. With this as backdrop, our goals here were to: (1) elucidate the perspectives and beliefs of key stakeholders on the scientific state of the art in, and potential for the clinical translation of, neuroimaging and mental health; and (2) to offer guidance based on the results for closing knowledge gaps, bridging hopes and tensions, and translating knowledge into action.

Our work is motivated by the central role that structural and functional neuroimaging have played in the search for a biological foundation for psychiatry. In 1976, Johnstone and colleagues demonstrated that patients with schizophrenia had enlarged cerebral ventricles [6], delineating for the first time the neural correlates of a psychiatric illness. In more recent years, neuroimaging researchers have used structural as well as functional signals to explore and discover the biological bases of mental illness.

A major focus of contemporary neuroimaging research is the identification of imaging biomarkers. Radioligands that bind to neurotransmitter receptors and transporters have been used to measure target occupancy for use in proof of mechanism studies, and to determine required dosage for new antipsychotic drugs [7] and anti-depressants [8]. Multivoxel pattern analysis has been used to develop imaging biomarkers for the detection of prodromal schizophrenia [9], and diffusion tensor imaging has been applied to the early detection of first episode psychosis in schizophrenia [10] and ASD in children [11].

Imaging genetics has also offered significant insights, allowing researchers to characterize the molecular pathways of neurological and mental health disorders [12]. Researchers working in this area have focused on three types of variants: functional variants with a known neurochemical effect [13,14]; common variants which are known to make a small or medium sized contribution to disease risk [15,16]; and rare variants which are known to make a large contribution to disease risk [17,18]. Substantial progress has also been made using genome-wide association studies (GWAS) that identify risk variants for psychiatric diseases in the absence of knowledge concerning their function. For example, several single-nucleotide polymorphisms on genes coding for subunits of the GABA-A receptor have been associated with bipolar disorder using GWAS [19].

Finally, imagers have begun to focus on simple behavioural states and traits associated with particular mental disorders. This research strategy is based on the supposition that it is easier to reliably identify the neural correlates of simple behavioural states and traits than the complex phenotypes typical of most mental disorders. This strategy has had success. For example, researchers working with patients with schizophrenia have used this approach to identify the neural activation patterns associated with verbal hallucinations by asking patients suffering from auditory verbal hallucinations to report the on- and offset of the voices [20-22].

Despite the importance of these breakthroughs, the overall progress in diagnostic and prognostic imaging biomarkers of mental disorders has been slower than anticipated. According to many experts in the field, diagnostic and prognostic imaging biomarkers – genetic or otherwise – remain a long way from clinical application [23-25].

Academic viewpoints notwithstanding, early adopters offer neuroimaging to mental health consumers on the open market in the USA and Canada. The homepage on the website of a prominent North American provider states:

*[We are] highly effective at helping people of all ages have better brains and better lives. Our success rate is very high by using detailed evaluations and brain SPECT [single photon emission tomography] imaging to better target treatment*[26].

Their website provides detailed information concerning how brain scans can help persons suffering from a variety of disorders. For example, the website says SPECT can specifically reach people with anxiety and depression by helping them to: evaluate whether or not the person has anxiety or depression, determine the type of anxiety or depression to inform treatment decisions, determine if treatment is effective, assess for the presence of co-occurring conditions, reduce emotional pain and stigma by demonstrating that symptoms and behaviors are not imaginary, increase treatment compliance, and gain a better understanding of mental illness [26].

Similar potential benefits – related to diagnosis, treatment selection, treatment monitoring, de-stigmatization, compliance, and understanding – are noted for persons with attention disorders, addictions, autism spectrum, behavioural problems, brain injury, marital conflict, memory issues, and weight issues [26]. To our knowledge, neither primary nor secondary insurance coverage is available for these services in any health system; customers pay out-of-pocket. The clinic quoted above offers customers “CareCredit” with 0% financing to enable prospective consumers to purchase neuroimaging services without delay.

The practice has drawn heated criticism from mainstream psychiatry. Although papers on the use of SPECT scans in psychiatry [27-32] have been published and providers claim that the work is “based on hundreds of texts and scientific articles” [33], critics contend that the publications do not support the use of SPECT imaging in psychiatric practice [34]. In support of this contention, critics cite the 2006 Practice Guidelines issued by the American Psychiatric Association [35] which state that:

… *the clinical utility of neuroimaging techniques for planning of individualized treatment has not yet been shown. Further research is needed to demonstrate a clinical role for structural and functional neuroimaging in establishing psychiatric diagnoses, monitoring illness progression, and predicting prognoses*[36].

A major review of neuroimaging in psychiatry published in the January 2012 issue of *Neuron* suggests that the APA’s position remains current. According to the author, neuroimaging

“… *so far has not yielded clinically relevant biomarkers for [disease, prognosis, or treatment of] mental disorders*” [23].

Objections to the direct-to-consumer approach, however, are not merely academic. Critics charge that the for-profit sector is jeopardizing public trust in the field and putting patients at risk. These charges are summarized in a 2010 letter published in the *American Journal of Psychiatry*:

*There are several dangers to patients that can accrue from [this] approach: 1) patients (including children) are administered a radioactive isotope without sound clinical rationale; 2) patients pursue treatments contingent upon an interpretation of a SPECT image that lacks empirical support; and 3) based on a presumed diagnosis…, patients are guided toward treatment that may detract them from clinically sound treatments*[34].

Although the battle lines in this acrimonious debate seem to be drawn, it is important to note that – to date – a systematic examination of the perspectives of key stakeholders involved has not yet been attempted. To this fill this void, we interviewed neuroimaging researchers, providers of neuroimaging services, and consumers of these services. Our goals were to: (1) elucidate the perspectives of researchers on the state of the art in translational neuroimaging research; (2) understand the opinions of providers currently offering functional neuroimaging clinically; and (3) explore the expectations and experiences of consumers of these services. From the triangulation of these findings, we offer guidance for translating of the resulting knowledge into action.

## Methods

This study was reviewed and approved by the behavioural research ethics board at the University of British Columbia. Informed consent was obtained from all participants. We conducted semi-structured interviews with: 1) investigators who conduct functional neuroimaging studies involving adults with mood disorders, schizophrenia, obsessive compulsive disorder, and/or attention deficit hyperactivity disorder; 2) providers who conduct or order functional neuroimaging examinations clinically in this population; and 3) consumers who, in order to obtain information related to mental, psychological, or emotional health, have purchased brain scans or have had brains scans purchased for them.

### Recruitment

#### Investigators

Academic investigators involved in functional neuroimaging research related to mood disorders (bipolar and unipolar disorders), schizophrenia, obsessive compulsive disorder (OCD), and/or attention deficit disorder (ADD)/attention deficit hyperactivity disorder (ADHD) who were fluent English speakers were identified using a combination of purposive and snowball sampling methods. We conducted two targeted searches of the USA-based National Institutes of Health (NIH) RePORTER database. The first search involved the search terms: “SBIR/STTR” AND “Functional neuroimaging” AND “Mental Health.” The second search utilized the search terms: “RO1 equivalents, DP2, RO1, R23, R29, R37” AND “functional neuroimaging” AND “mental health.” We also conducted a parallel search of the Canadian Institutes of Health Research (CIHR) funded research database using the search terms: (any of the phrases) mood, bipolar, anxiety, schizophrenia AND (any of the phrases) imaging. An initial set of prospective participants was created from the search returns. Additional participants were identified via snowball sampling.

#### Service providers

Clinicians working at clinics that offer clinical neuroimaging services related to mood disorders (bipolar and unipolar disorders), schizophrenia, OCD, and ADD/ADHD were also identified using purposive and snowball sampling. We created an initial set of potential participants by searching the Internet for clinicians who satisfied the above eligibility criteria. We then used snowball sampling to identify additional eligible participants not identified in the initial search.

#### Consumers

Men and women age 19–75 who, in order to obtain information related to mental, psychological, or emotional health, had purchased brain scans or had had brain scans purchased for them, who had the capacity to understand the nature of the study, who could provide verbal informed consent, and who are fluent in English were identified through one of four recruitment strategies: (1) Google ad linked to key words “brain MRI imaging,” “SPECT,” MRI scan of the head,” “brain scan results,” brain MRI scan,” and “cost of brain MRI scan,” with a one-line description of the study, and a link to a recruitment poster posted on the homepage of the authors’ organizational website; (2) Craigslist ad (San Francisco) with a one-line description of the study, and a link to the homepage of the authors’ organizational website; (3) ads on five top rated mental health websites as rated by the analytic site ‘compete.com’ at the time of recruitment, with a one-line description of the study, and the link to the recruitment poster; and (4) poster on the website or in the newsletter of service providers.

### Data collection and analysis

Data for this study constituted open answers to questions posed during semi-structured interviews designed to take approximately 30 min. All interviews were conducted over the telephone. Verbal consent was obtained and all interviews were audio-recorded. Confidentiality was achieved by assigning an alphanumeric identifier to each participant and immediately disassociating names from responses.

Interviews of investigators and service providers followed the same interview guide. Questions focused on a range of salient issues including the focus of their research on the continuum from basic research to clinical application, challenges, and the future of functional neuroimaging research. The questions are summarized in Table 1. This table also summarizes the parallel but separate set of questions developed for consumers.

**Table 1:** Summary of interview questions

**Investigators and service providers**	**Consumers**
• Location of research on a continuum from basic research to clinical application	• Sources of information about functional neuroimaging
• Focus of efforts	• Features of interest in about brain scans
• Challenges faced in research and practice	• Motivation to pursue a brain scan
• Barriers to clinical translation of functional neuroimaging	• Knowledge gained
• Necessity of prospective clinical trials	• Impact of experience
• Risks and benefits to patient-participants	• Expectations and level of satisfaction with experience and brain scan

### Analysis

All interviews were transcribed and made software ready (NVivo 9, QSR International). We applied the qualitative methodology of constant comparison to analyze the data, whereby two researchers independently examined the transcriptions. This involved segmenting raw data, labelling and coding, while searching for patterns of intersection. Major themes and their constituent subthemes were counted only once even if they appeared more than once in a given transcript to provide control for individual biases. We applied an interpretative and iterative process, critically analyzing and conceptualizing the results into textual themes [37-41]. Coding discrepancies were highlighted and discussed between two trained coders to achieve consensus. Dominant themes and subthemes emerged from the triangulation of all aspects of the analysis. Quotations presented in Results below were chosen for their representativeness of the phenomena identified and described.

## Results

Tables 2, 3 and 4 shows the full range of themes and subthemes derived from the analysis, and their distribution and frequency of occurrence across interviews with investigators, providers, and consumers. We present prominent themes as they emerged from the coding strategy. Full details of the constituent subthemes are shown in the tables. We provide illustrative quotations in the text below to enrich the data.

**Table 2:** Interviews with investigators

**Coded themes/Subthemes**	**Number of interviews in**	** Coded themes/Subthemes**	**Number of interviews in**
	**which coded themes and**		**which coded themes and**
	**subthemes occur/total N**		**subthemes occur/total N**
	**(N = 28)**		**(N = 28)**
**Goals of research**		**Risks to participants**	
Treatment choice	9	Noise, claustrophobia	11
Prediction	7	Incidental findings	9
Monitoring	5	False hope for treatment	8
Treatment	4	Distrust of science or medicine	3
Diagnosis	3	Inaccurate diagnosis	6
Pre-surgical planning	3	Increased stigma	5
Defining populations	1	Negative self-understanding	4
**Challenges**		Distrust of science or medicine	3
Scientific challenges	12	Breach of confidentiality	2
Funding challenges	8	Discrimination (e.g., insurance)	2
Technical limitations	5	None (explicitly)	3
Heterogeneous protocols	1	**Future of neuroimaging**	
Participant recruitment	4	Clinical trials needed	16
Ethics review itself	2	Clinical trials not needed	1
Balancing research with care	2	Larger samples	13
Obtaining informed consent	1	Multi site trials	12
Lack of creativity in the field	2	Controlled studies	7
**Potential benefits**		Blinded trials	5
Improved care	9	Randomized trials	5
Better understanding illness	6	Longitudinal studies	2
Reduced stigma	5	Add-on studies	2
Improved self-attitude	1	Standardized imaging protocols	6
New knowledge	2	Different models of disease	2
None (explicitly)	2	Drug trials	2
		**Clinical Uptake**	
		Demand from clinicians	1
		Science not ready	11

**Table 3:** Interviews with service providers

**Coded themes/Sub-themes**	**Number of interviews in**	** Coded themes/Sub-themes**	**Number of interviews in**
	**which coded themes and**		**which coded themes and**
	**subthemes occur/total N**		**subthemes occur/ total N**
	**(N = 5)**		**(N = 5)**
**Goals of practice**		**Potential benefits**	
Treatment choice	4	Improved care	4
Treatment	2	Reduced stigma	3
Diagnosis	3	Increased family support	1
Compliance	1	Improved self-attitude	1
**Challenges**		**Risks to patients**	
Lack of creativity	1	False hope for treatment	2
Professional censure	1	Cost	1
Lack of clinician-researchers	1	Misuse of technology	2
Limited clinical knowledge	1	Patient self-understanding	1
Symptom-based diagnosis	2	**Future of neuroimaging**	
Distrust of the Academy	1	Clinical trials needed	1
Limited training	1	Clinical trials not needed	1
Lack of clinical interest	1	Controlled studies	1
Insurance	1	Standardized imaging protocols	1
		**Rationale for clinical use**	
		Demand from other clinicians	1
		Scientific promise	1

**Table 4:** Interviews with consumers

**Coded themes/Subthemes**	**Number of interviews in**	** Coded themes/Subthemes**	**Number of interviews in**
	**which coded themes and**		**which coded themes and**
	**subthemes occur/total N**		**subthemes occur/ total N**
	**(N = 28)**		**(N = 28)**
**Source of information**		**Impact of scan**	
Popular media	20	Change of treatment	18
Professional sources	5	Better treatment	10
Word of mouth	4	Relief or quality of life	18
**Motivation to seek scan**		Better understanding of disorder	14
Objective diagnosis	23	Improved self-attitude	13
Better understanding	14	Sense of empowerment	11
Better treatment	11	Acceptance by others	11
Skepticism - psychiatry	11	Increased hope	10
Undiagnosed concerns	8	Belief in diagnosis	6
No other options	5	Decreased stigma	5
Legal reason	2	Decreased hope	4
**Concerns about the scan**		None (explicitly)	2
Cost	13	Worse self-attitude	1
Radiation	6	No change in treatment	1
Meaningfulness	4	Decreased compliance	1
Unexpected bad news	3	**Evaluation**	
Lack of insurance	3	Expectations fully met	22
Claustrophobia	2	Would do it again	22
Uninformative findings	2	Worth the cost	8
Confidentiality	1	Expectations partly met	5
Change in therapy	1	Would not do it again	1
**Results received**		**Overall experience**	
New primary diagnosis	12	Positive	25
Confirmation of existing diagnosis	11	Negative	3
New secondary diagnosis	11		
Exclusion of a suspected diagnosis	2		
None (explicitly)	2		

### Investigators

Altogether thirty-six investigators were invited by email to participate. Twenty-two investigators completed the study, at which point recruitment was terminated due to theoretical saturation [39,40] of the data. Twenty of the twenty-two investigators viewed their work in at least partly translational terms on the spectrum from bench research to bedside care, with two describing their work in purely basic or non-translational terms. The investigators in the former group located their work at various, sometimes overlapping stages on the translational pathway. Eighteen of these investigators viewed their work as preparatory to clinical studies proper – what we called “developing tools for clinical translation.” Ten of the investigators reported that they were participating in clinical studies.

Six major themes emerged as determined by their prevalence in the discourse of the participants interviewed (Table 2):

(1) Goals: Improving and facilitating treatment choice for people with mental illness emerged as the dominant code under the theme of goals. For example,

The current purpose of my work is to understand brain mechanisms of how people get better from depression and anxiety… There are two purposes for this. One is to create personalized treatment algorithms. That is to better refer people to treatments that they would likely benefit from. And two, to help to refine the treatments that exist, or create treatments that more effectively target brain mechanisms associated with disease which are not targeted in current treatments (NII007).

(2) Challenges: Investigators identified a broad range of challenges associated with research in the area of neuroimaging for mental health disorders. Limitations related to the state of the science was the most prominent code. For example,

I just don’t think that the science is there yet … (NII008).

(3) Benefits: Improved care was the code that emerged prominently under the theme of benefits. Emergent codes also highlighted the importance of possibilities for a better understanding of mental illness and de-stigmatization. For example,

… Neuroimaging has] had an important contribution to kind of the sociology of how we think about mental illness. (NII05).

(4) Risks: Noise, claustrophobia, and radiation were the risks that emerged prominently in the data. Risks of incidental findings, distrust of medicine, discrimination, and breaches of confidentiality were also coded.

(5) Future of functional neuroimaging: The need for clinical trials was a dominant code in response to the question about the future of the science. For example,

We have been tremendously, tremendously limited by small sample sizes, by particular oddities of particular populations or treatments, and lack of an understanding of what a placebo does, for example, relevant to a medication. Or even what a real clinical choice might be that a person—a provider with a patient might be faced in the clinic, and aligning that with how we do our research. So putting functional neuroimaging with actual, properly done clinical trials helps merge those sets of interests and challenges (NII008).

(6) Clinical uptake: The immaturity of imaging science in the context of mental health and the consequent need for caution with respect to clinical application, characterized the dominant response to the question about readiness of the technology for the psychiatry clinic. For example,

… imaging is not as strong for defining diseases as the DSM criteria are… it’s not as accurate as standard behavioural interviews and scales for diagnostic classification… I just think we’re not doing it because it’s not a responsible thing to do… (NII012).

### Service providers

We identified eleven eligible participants who satisfied the eligibility criteria for the study. Five participated. These participants saw their work in clinical terms, with four of the five providers stating that they were actively using functional neuroimaging in their clinical practice. One provider also noted involvement in clinical research.

The major themes coded from this group were similar to those emerging in the interviews with investigators (Table 3):

(1) Goals: Treatment choice was the dominant code in the discourse about goals. The use of functional neuroimaging for diagnostic purposes was also prominent in the interviews.

(2) Challenges: Limitations of symptom-based diagnostic categories was the most frequent code concerning the challenges associated with the use of neuroimaging in clinical psychiatry. For example,

[W]hat’s going on in the country is that there’s a very significant limitation to the current diagnostic coding system. It’s really, to be completely plain about it, based on appearances, because really all we’ve had for years is appearances. Now, I mean there are some diagnostic codes in there that having something to do with genetics… [T]he bottom line is, human beings in the office are labelled by how they behave, and by what an external person thinks their behaviour is, as opposed to what’s actually necessarily going on. And so imaging’s a very important point. If you’re using—if you’re finding that using the current diagnostic naming system to drive your treatments is limited and insufficient, it’s going to be natural to seek further information with more brain function evidence (NII026).

Distrust and professional censure, limited training opportunities, lack of creativity in the field, and a shortage of medically-trained neuroimaging researchers were also coded.

(3) Benefits: Benefits related to the possibilities for improved care and the destigmatization of mental illness were the primary codes related to benefits in the interviews of providers.

(4) Risks: False hope and the misuse of technology were the most prominent risk codes. For example,

The risk of the test is misusing the test. The risk is telling—giving people false hope, which I—which I never do. I think it’s very important to explain to patients as carefully as possible, like, what the benefits, what the risk –, what I can do with the information, what I can’t do (NII032).

(5) Future of neuroimaging: Coding highlighted an emphasis on guidelines and standardization but not necessarily on clinical trials. For example,

[W]hat do we need to make it more possible to have imaging really a standard part of psychiatric medicine? …we need standards that we don’t have. Standard ways to do the procedure, to interpret the procedure, and a standard body of literature that everybody can refer to (NII022).

(6) Clinical uptake: When asked why functional neuroimaging was limited clinically in the context of mental illness, providers cited a number of factors including lack of interest. The following quotation is illustrative:

There’s not enough people who are doing it that are excited about it. It’s not that it’s not useful. (NII022).

### Consumers

Seventy-seven individuals responded to the recruitment ads for the consumer arm of the study. Most of these individuals learned about the study through posters in the offices, newsletters, or websites of service providers. The rest accessed information about the study via our Google Ad or web-based mental health fora. There were no respondents to the advertisement on Craigslist. Of the seventy-seven individuals who responded initially, six were ineligible and forty-three did not respond to follow up. Twenty-eight respondents constituted the final sample, yielding a response rate of 44%.

All participants completed a demographic survey before the interviews began. The majority of participants were Caucasian (27 of 28), and one was African American. A majority of the participants were female (17 of 28) Almost sixty percent of the participants reported incomes over $75K USD, and forty-three percent of respondents reported that they held a graduate degree.

Seven major themes were coded from the interviews with consumers (Table 4):

(1) Sources of information about brain imaging: Popular media (i.e., Internet, television, popular books) emerged as the primary source of information.

(2) Motivation: Coding highlighted consumers’ desire for a clear and objective diagnosis as the primary motivation for pursuing neuroimaging in the open marketplace. For example,

… I thought that with certainty, the kind of certainty you can get with a picture, maybe that would help us… (C007).

… brain imaging would get us a better assessment, a more complete assessment than the nonsense I was getting here (C024).

Hope for better treatment and distrust of traditional psychiatry also emerged prominently in the coded interviews.

(3) Concerns: Little in the data suggested concerns about neuroimaging. The few coded units for this theme related to radiation.

(4) Results received: New diagnosis, confirmation of an existing diagnosis, or a new secondary diagnosis dominated codes for results received. For example,

[T]hey said I had kind of an ADD process where, you know, when I’m concentrating, especially on, you know, something that’s relatively mundane, there’s a decrease of function in the front part of my brain, so that it lends itself to having more difficulty with staying on task with boring things (C014).

(5–7) Impact, evaluation and experience: Consumers identified many types of impact from the clinical consultation they received. Changes in treatment and physical or psychological relief dominated the codes for this theme. For example,

I was relieved to know that it was not – that I wasn’t crazy (C039).

[T]his made me feel more in control of my own life. And that’s a really good feeling to have. That’s definitely worth thirty-five hundred dollars (C023).

Better understanding of mental illness, improved self-attitude, a sense of empowerment, and increased hope were also coded frequently. We coded a few instances of negative discourse regarding decreased hope, impeded compliance with treatment, and a worsened attitude toward the self.

## Discussion

To map the current translational landscape of functional neuroimaging for mental illness, we interviewed investigators who are conducting functional neuroimaging studies involving adults with mood disorders, anxiety disorders, or schizophrenia, providers who are applying functional neuroimaging clinically in this population, and consumers of these services.

We found six to seven major emergent themes in each group, and among them convergence in perspectives across the three groups in some domains. Investigators, service providers, and consumers held common perspectives about the potential or actual risks and benefits of functional neuroimaging for mental illness. All three groups pointed to improved care as a major goal of functional neuroimaging in this context and shared similar goals for improved diagnosis, treatment choice, and compliance. All three groups also discussed cost and access, the importance of mitigating false hope, and the meaningfulness of findings.

These convergences notwithstanding, the divergences in perspectives between participants in the three groups are striking if not unexpected. When asked about the challenges they face in their work, the concerns of investigators and service providers overlapped to a limited extent. Overall, the persistence of symptoms based diagnostic categories was the challenge noted most frequently by service providers, whereas the limitations of the science in this area was the challenge noted most frequently by investigators.

Consumers’ perspectives on their own experiences with brain scans sit uneasily against the backdrop of these professional disagreements. Though some consumers did express concerns about the validity or efficacy of functional neuroimaging in this context, consumers’ evaluation of the service they received was positive: the majority stated their expectations were met. That said, only a subset stated they would do it again or recommend it to others given the $3500-4000 USD cost.

In sum, the evidence paints a conflicted picture. Academic investigators are skeptical about the clinical application of neuroimaging in the context of mental health given the current state of the science. By contrast, commercial service providers are enthusiastic, based on their clinical experience for the most part. Consumers, finally, are caught in the middle. All told, this scenario poses dangers to everyone: academic investigators risk missing the opportunity to shape the uptake of this emerging technology in the marketplace; commercial service providers, who already face significant resistance, risk a backlash from the academic community; and consumers themselves may come to distrust both parties.

Indeed, such open antagonism between the academic and commercial communities undermines trust in science and medicine and, ultimately, does a disservice to the public. As Ellison writes in her recent book *Buzz* (2010), we are

… drowning in data and confused by endless controversies (p. 149).

Professional disputes may be newsworthy but they are often unproductive distractions as well.

It is important to note the limitations of this study. The data reflect the perspectives of a closed and limited set of investigators, providers, and consumers. The service provider pool is particularly small, and the use of snowball sampling to identify investigators and service providers may have biased the results. Consumers who self-selected to participate may have been particularly satisfied with services received. The data are also limited in terms of the overall ethnic and cultural diversity of participants. Finally, some degree of coder bias is inevitable when working with qualitative data, even in the face of systematic analyses and consensus.

Study limitations notwithstanding, these findings shed light on the current translational landscape of functional neuroimaging for mental illness. It is clear that much work needs to be done to both minimize existing tensions and to maximize the potential of this exciting new technology. With these goals in mind, we draw upon the data to offer the following recommendations for future action:

### Create a mental health neuroimaging consortium

We recommend the creation of a consortium for functional neuroimaging in mental health, that builds on the strengths of existing shared databases such as *BRAINNet, OpenfMRI, BrainMap,* meta-analytic methods such as the *ALE* and *ES-SDM*, and others such as the Alzheimer's Disease Neuroimaging Initiative (ADNI). Members of the consortium would follow standardized protocols for data acquisition, data analysis, and repositories for data mining and sharing. It should be co-funded by major government health agencies that have mental health at the focus of their mandate, and properly include a component dedicated to cultural representation. The consortium should focus initially on biomarkers for treatment selection and monitoring in a discrete number of disorders. Given advances particularly in the area of schizophrenia [23], this would be a good first target. The consortium should partner with the pharmaceutical industry to conduct large clinical trials akin to drug and cancer trials [25]. These should be unencumbered and monitored by an independent scientific board. The work should be conducted within an open and transparent framework of collaboration that destigmatizes academy-industry research and mitigates suspicion of bias.

### Conduct retrospective studies of extant databases

We recommend retrospective studies of extant commercial image databases. The for-profit imaging sector boasts vast image databases but, to date, they remain an untapped resource. Studies of these databases could be made possible via open competition for research contracts and paid for in part by the commercial sector and carried out in partnership with academia. These studies would need to undergo continuous review by a scientific advisory board that has equal membership from both sectors. Rigorous methods and effective dissemination strategies anchored in pre-engagement ethics agreements should be central requirements of potential proposals.

### Build on commercial reach and know-how

The academic neuroimaging community, academic institutions, research sponsors, and others should harness the know-how and reach of the commercial marketing machine to raise awareness and improve education of mental health disorders. The visibility of the commercial sector is indisputable. With careful management, there is an opportunity to better promote mental health and well-being using these methods.

## Conclusion

Our findings point toward a fundamental tension between academic investigators on the one hand, and commercial service providers and their customers on the other. This scenario poses dangers to the communities directly involved, and to public trust in science and medicine more generally. Much work needs to be done to mitigate these dangers and maximize the potential of exciting new technology. With a focus on the patient and the collective strengths of both the research and service provider communities, constructive steps can be successfully undertaken to achieve these parallel goals.

## Competing interests

The authors declare that they have no competing interests.

## Authors’ contributions

JA, AM, and JI designed the study. JA conducted the interviews. JA and AM coded the data. JA, AM, and JI analyzed the results. JA wrote the first draft of the paper. JI reviewed the draft. JA, AM, and JI approved the final draft of the paper. All authors read and approved the final manuscript.

## Pre-publication history

The pre-publication history for this paper can be accessed here:

http://www.biomedcentral.com/1471-244X/13/208/prepub
